# Physical and Mental Health of Nurses During COVID-19: A Pilot Study on the Role of Work Engagement and Musculoskeletal Symptoms

**DOI:** 10.3390/epidemiologia6040093

**Published:** 2025-12-18

**Authors:** Luciano Garcia Lourenção, José Gustavo Monteiro Penha, Daniela Menezes Galvão, Luiz Antônio Alves de Menezes Júnior, Daiani Modernel Xavier, Natália Sperli Geraldes Marin dos Santos Sasaki, Francisco Rosemiro Guimarães Ximenes Neto, Jacqueline Flores de Oliveira, Alberto de Oliveira Redü, Max dos Santos Afonso, Vagner Ferreira do Nascimento, Rita de Cássia Helú de Mendonça Ribeiro, Renato Mendonça Ribeiro, Daniele Alcalá Pompeo, Sidiane Rodrigues Bacelo

**Affiliations:** 1 School of Nursing, Federal University of Rio Grande, Rio Grande 96200-400, Brazil; gustavo_penha02@hotmail.com (J.G.M.P.); dani.mgalvao@hotmail.com (D.M.G.); daiamoder@gmail.com (D.M.X.); betoredu@hotmail.com (A.d.O.R.); sidiane.rodrigues.enfermeira@gmail.com (S.R.B.); 2 Minister’s Office, Ministry of Social Security, Federal Government of Brazil, Brasília 70059-900, Brazil; 3 University Hospital of the Federal University of Sergipe, Brazilian Hospital Services Company (EBSERH), Aracajú 49060-025, Brazil; 4 School of Nutrition, Federal University of Ouro Preto, Ouro Preto 35400-000, Brazil; alves.luizantonio@gmail.com; 5 Department of Specialized Nursing, Faculty of Medicine of São José do Rio Preto, São José do Rio Preto 15090-090, Brazil; nsperli@gmail.com (N.S.G.M.d.S.S.); ritadecassia@famerp.br (R.d.C.H.d.M.R.); daniele.pompeo@famerp.br (D.A.P.); 6 Health Sciences Center, Vale do Acaraú State University, Sobral 62042-280, Brazil; rosemironeto@gmail.com; 7 Faculty of Medicine, Catholic University of Pelotas, Pelotas 96010-901, Brazil; jacqueoliveira.enf@hotmail.com (J.F.d.O.); max.afonso@hotmail.com (M.d.S.A.); 8 Indigenous Intercultural College, Mato Grosso State University, Barra do Bugres 78390-000, Brazil; vagnerschon@hotmail.com; 9 Department of Science and Technology, Brazilian Army, Brasilia 70630-900, Brazil; drrenatoribeiroenf@gmail.com

**Keywords:** nursing, work engagement, compassion fatigue, musculoskeletal symptoms, occupational epidemiology

## Abstract

Background/Objectives: Nursing professionals were among the most affected groups during the COVID-19 pandemic, exposed to simultaneous physical demands and emotional strain. This study examined the interplay between work engagement, compassion fatigue, and musculoskeletal symptoms among frontline nurses in a Brazilian public hospital. Methods: A cross-sectional study (*n* = 77) was conducted between February and April 2022 using validated instruments (Work Stress Scale, ProQoL-BR, Nordic Musculoskeletal Questionnaire, and UWES-9). Descriptive and inferential analyses were performed (*p* ≤ 0.05). Results: Most participants did not report occupational stress (84.4%). No profiles of compassion fatigue were identified, although notable rates of burnout (26.0%) and secondary traumatic stress (23.4%) were observed. Engagement scores were very high in vigor and dedication. Musculoskeletal symptoms were prevalent, especially in the lumbar region (chronic: 60.0%). Female sex, statutory employment, and lack of physical activity were associated with a higher prevalence of symptoms and sick leave. Work engagement (vigor and overall score) showed negative correlations with absenteeism. Conclusions: The coexistence of high engagement and emotional vulnerability, in the absence of compassion fatigue, suggests that higher levels of engagement may be associated with lower occupational stress. These findings highlight the importance of integrated strategies, including ergonomic interventions, health promotion, and organizational support, to preserve the physical and mental health of frontline nursing professionals. This study provides new evidence of engagement as a potential protective factor that may mitigate physical and emotional burden among nurses in resource-limited settings.

## 1. Introduction

The COVID-19 pandemic has imposed unprecedented challenges on healthcare systems and the global workforce. Nursing professionals, due to their direct and continuous frontline involvement, were exposed to multiple physical, emotional, and occupational risks, becoming one of the groups most affected by the crisis [1,2].

International studies have reported a high prevalence of mental health–related symptoms among these workers, including anxiety, depression, emotional exhaustion, and compassion fatigue [3,4,5,6]. In parallel, physical overload resulting from long working hours, insufficient rest periods, and ergonomically unfavorable practices has increased the occurrence of musculoskeletal symptoms, such as back, neck, and shoulder pain [7,8].

Occupational stress, one of the primary psychosocial risks in nursing, is defined as a state of tension that, when persistent, triggers psychosomatic manifestations such as cardiovascular changes, gastritis, sleep disturbances, and irritability. The classic model of stress adaptation describes three phases: alert, resistance, and exhaustion—the latter characterized by severe physical and mental impairment and reduced professional performance [9,10]. Nursing is particularly vulnerable to this process, given its combination of direct patient care with organizational and administrative responsibilities [10,11].

The intensification of physical overload during the pandemic contributed to the development of Work-Related Musculoskeletal Disorders (WMSDs), especially in the lumbar spine, shoulders, and neck, leading to persistent pain, absences from work, and compromised health [12]. At the same time, compassion fatigue emerged as a form of cumulative emotional exhaustion due to continuous exposure to patient suffering, and is associated with burnout, secondary traumatic stress, and reduced motivation and empathy [13,14,15,16,17].

Furthermore, recent evidence indicates that burnout has intensified among nurses during the pandemic, driven by imbalances between effort and reward, emotional overload, and deteriorating working conditions. In a study with nurses and midwives in Lebanese hospitals, Sayrafi et al. found that high levels of burnout were associated with greater occupational stress and reduced adherence to standard precautions, highlighting how professional exhaustion compromises both mental health and patient safety in crisis contexts [18]. These findings underscore the importance of examining burnout in an integrated manner alongside other psychosocial factors affecting nursing professionals during COVID-19.

In contrast, work engagement has been described as a protective factor. This positive psychological state, comprising vigor, dedication, and absorption, is associated with resilience, well-being, and improved performance [5,16,19]. Supportive organizational environments tend to foster engagement, thereby buffering the effects of stress and emotional fatigue [2]. Studies in diverse healthcare settings, including Brazilian university hospitals, have reported high levels of dedication and vigor coexisting with anxiety or depression, suggesting that engagement may help preserve mental health even under adverse conditions [6,20].

Thus, during the pandemic, a duality became evident: a high risk of illness alongside the possibility of finding in engagement a resource to buffer overload and sustain mental health, with implications for worker well-being, patient safety, and the sustainability of health services [4,21]. Despite extensive literature on the pandemic, few studies have integrated physical and emotional dimensions in a comprehensive analytical framework, particularly in middle-income countries with resource constraints and precarious labor relations [10,21,22]. Moreover, engagement remains insufficiently explored within such integrative models [23].

Based on this gap, we hypothesized that frontline nursing professionals experienced significant physical and mental health impairments, but that engagement played a protective role in mitigating occupational stress. Therefore, this study examined the interplay between work engagement, compassion fatigue, and musculoskeletal symptoms among frontline nurses in a Brazilian public hospital.

## 2. Materials and Methods

### 2.1. Study Design, Period, and Setting

This was a census-based, cross-sectional, descriptive, and correlational study conducted between February and April 2022 in the Urgent Care and Emergency Department and the COVID-19 Ward of the Dr. Miguel Riet Correa Júnior University Hospital of the Federal University of Rio Grande (HU/FURG), located in the city of Rio Grande, Rio Grande do Sul, Brazil.

Data collection occurred during a period of intensified epidemiological activity of COVID-19 in Brazil. Between January 2020 and March 2023, the country recorded more than 38 million confirmed cases and over 700,000 deaths. In the state of Rio Grande do Sul, more than 3.1 million cases and 43,000 deaths were documented, while the city of Rio Grande reported approximately 62,000 cases and 740 deaths. During this period, HU-FURG played a central role in the regional response, expanding intermediate and intensive care beds dedicated to COVID-19, implementing a molecular diagnostic laboratory capable of processing up to 100 tests per day, and establishing a Post-COVID-19 outpatient clinic.

During the pandemic, the hospital reorganized its emergency department into separate wings for COVID-19 and non-COVID-19 patients and created a dedicated ward with 18 intermediate and semi-intensive care beds.

### 2.2. Population and Sample

The study population consisted of 85 nursing professionals (24 nurses and 61 assistants/technicians). Inclusion criteria were employment at the institution for at least two months, considered the average adaptation period to the organizational routine [24], and active work in the studied sectors during the pandemic. Professionals reassigned due to health-related risk conditions and those with temporary contracts were excluded to ensure sample homogeneity and minimize bias associated with short-term employment and weaker institutional ties.

The final sample comprised 77 participants (90.6% of the population): 23 nurses (29.9%) and 54 assistants/technicians (70.1%). This high response rate, covering more than 90% of the target population, enhances sample representativeness and reduces the likelihood of selection bias, thereby strengthening the internal validity of the study.

### 2.3. Study Protocol

Data were collected in person by a trained nurse. After clarification and informed consent, participants completed self-administered questionnaires, which were returned anonymously by the following shift. When professionals were unable to respond during working hours, the deadline was extended to the subsequent shift. Five instruments were applied.

Sociodemographic and occupational questionnaire (developed by the authors), which included: professional category, age, sex, marital status, education, income, employment type, years of experience, work shift, sleep duration, additional jobs, and self-reported physical activity. The questionnaire underwent a pilot test with a comparable group of professionals to ensure clarity and comprehension. These individuals were not included in the final sample.

Work Stress Scale (WSS), validated by Paschoal and Tamayo [25], composed of 23 items rated on a five-point Likert scale (1 = strongly disagree to 5 = strongly agree). Average scores ≥ 2.5 indicate significant stress [25]. The variable was classified dichotomously as “with stress” (WSS ≥ 2.5) or “without stress” (WSS < 2.5). In this study, the Cronbach’s alpha for the Work Stress Scale (WSS) was 0.91, indicating excellent reliability.

Professional Quality of Life Scale–Brazilian version (ProQoL-BR), comprising 30 items measuring compassion satisfaction, burnout, and secondary traumatic stress [26]. Responses are given on a five-point Likert scale ranging from 0 (never) to 5 (almost always). Subscale scores were calculated and subsequently standardized into t-scores [27]. Compassion fatigue was defined as the simultaneous presence of high burnout and high secondary traumatic stress (≥75th percentile), consistent with prior empirical studies [16,28]. In our study, the Cronbach’s alpha coefficients for the three dimensions of ProQoL-BR demonstrated adequate internal consistency: Compassion Satisfaction (α = 0.84), Burnout (α = 0.70), and Secondary Traumatic Stress (α = 0.73).

Nordic Musculoskeletal Symptom Questionnaire (NMSQ), translated and adapted by Pinheiro et al. [29], used to assess symptoms in 10 body regions over the past 7 days and 12 months, including absences due to musculoskeletal symptoms within the previous 12 months. Symptoms were quantified based on the frequency of complaints in the specified periods and on reported absences attributed to musculoskeletal causes. The Cronbach’s alpha for the NMSQ in our study was 0.82, demonstrating good internal consistency.

Utrecht Work Engagement Scale (UWES-9), containing nine items distributed across the dimensions of vigor (three items), dedication (three items), and absorption (three items) [30,31]. Items are rated on a seven-point Likert scale (0 = never to 6 = always). Subscale scores were calculated as arithmetic means and classified into established reference categories: Very Low (0–0.99), Low (1–1.99), Moderate (2–3.99), High (4–4.99), and Very High (5–6) [30]. In the present study, the Cronbach’s alpha coefficients to UWES-9 demonstrated adequate reliability for all dimensions: dedication (α = 0.77), absorption (α = 0.70), and vigor (α = 0.77), with excellent internal consistency for the overall score (α = 0.80).

### 2.4. Data Analysis and Statistics

Data were double-entered in Microsoft Excel^®^ for Microsoft 365 (Microsoft Corp., Redmond, WA, USA) and verified using Data Compare^®^, module of EpiData software, version 4.6 (EpiData Association, Odense, Denmark), then analyzed in SPSS^®^ version 25.0 (IBM Corp., Armonk, NY, USA). Normality was assessed using the Kolmogorov–Smirnov test. Descriptive statistics and inferential analyses (Pearson’s correlation and Student’s *t*-test; *p* ≤ 0.05) were applied. Correlation coefficients were classified as weak (r ≤ 0.30), moderate (0.40–0.60), or strong (r ≥ 0.70).

We attempted to fit multivariate models (logistic and Poisson regression) to adjust for potential confounders such as age, sex, professional experience, employment type, work shift, and physical activity. However, these models exhibited non-convergence, unstable coefficients, and excessively wide confidence intervals, issues likely related to the limited sample size, low outcome frequency, and separation problems commonly observed in small datasets [32,33]. Due to these limitations, multivariate analyses were not retained, and results were based on robust bivariate procedures.

Covariates used in stratified or bivariate analyses included sex (male/female), age (26–35 or ≥36 years), family income (2–5 or 6–10 minimum wages), professional category (nurse/assistant–technician), employment type (Consolidation of Labor Laws [CLT in Portuguese/statutory), work shift (day/night), professional experience (<10/≥10 years), daily sleep (<6/6–8 h), and physical activity (yes/no).

For the evaluation of musculoskeletal symptoms, a sum score was calculated for each category. Acute symptoms were defined as pain, discomfort, or numbness occurring within the previous seven days; chronic symptoms were defined as symptoms present within the previous 12 months; and absences were defined as the need to refrain from normal activities (work, household tasks, or leisure) due to musculoskeletal symptoms within the previous 12 months. Scores ranged from 0 to 10, where “0” indicated the absence of symptoms and “10” indicated symptoms in all 10 evaluated regions.

## 3. Results

The sample consisted of 77 nursing professionals, 29.9% of whom were nurses and 70.1% nursing assistants or technicians. Most participants were female (68.8%) and aged between 36 and 50 years (45.5%), corresponding to the period of professional maturity according to the classification of Machado et al. [34]; 20.8% did not report their age. Regarding marital status, 58.4% were married or in a stable union, and 29.9% were single. In terms of education, there was a predominance of participants with a high school diploma (66.2%), followed by those with higher education (33.8%). Most were employed under the Consolidation of Labor Laws-CLT (83.1%) and reported a family income between two and five minimum wages (57.1%).

In terms of professional practice, 96.1% worked directly in healthcare, and the night shift was the most frequent (53.2%), followed by the full day shift (20.8%). Approximately 20.8% reported having another paid job. Most had more than 10 years of professional nursing experience (61.0%), although almost half (49.4%) had between two and five years of experience at the university hospital. Regarding health-related habits, 58.4% did not engage in regular physical activity and 63.6% reported sleeping between six and eight hours per night.

The analysis of mental health conditions showed that most professionals (84.4%) did not experience occupational stress, suggesting a scenario of relative emotional stability in the face of work demands. Regarding compassion fatigue, substantial proportions of professionals presented high scores for burnout (26.0%) and secondary traumatic stress (23.4%). However, no participant simultaneously exhibited high levels of burnout and secondary traumatic stress—a necessary condition to characterize compassion fatigue. These findings indicate that, although some signs of emotional exhaustion were reported, no critical pattern of emotional impairment associated with caregiving was identified in the study setting.

The assessment of work engagement (Table 1) revealed high scores across all dimensions. Dedication, vigor, and overall engagement scores were classified as very high, whereas absorption was classified as high, indicating strong involvement and commitment among nursing professionals.

Regarding musculoskeletal symptoms (Table 2), the lumbar region was the most affected (37.3% for acute symptoms, 60% for chronic symptoms, and 26.3% for sick leave), followed by the shoulders and neck. Body regions such as the hips, elbows, and forearms showed lower prevalence.

**Figure 1:**
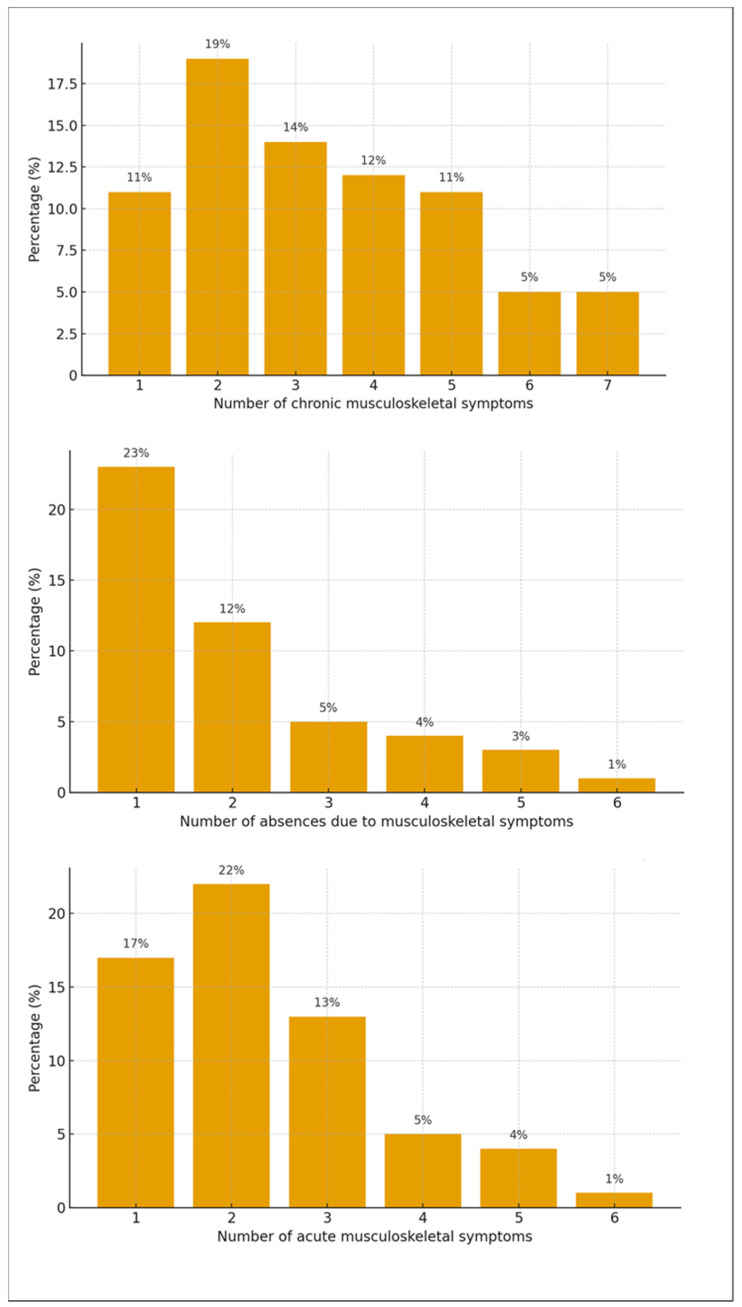
Percentage of occurrence of acute and chronic musculoskeletal symptoms and absences among nursing professionals.

The number of acute symptoms reported by nursing professionals ranged from zero to six (mean = 1.55; SD = 1.54); chronic symptoms ranged from zero to seven (mean = 2.73; SD = 2.10); and absences ranged from zero to six (mean = 1.04; SD = 1.43). Figure 1 presents the percentage distribution of nursing professionals according to the number of symptoms reported (acute, chronic, and related absences).

The bivariate analysis (Table 3) identified significant differences according to sex (women reported more acute symptoms; *p* < 0.001), employment type (CLT employees had a higher mean number of symptom-related absences compared to statutory workers; *p* = 0.043), and physical activity (sedentary professionals reported more chronic symptoms; *p* = 0.028). No significant associations were found for age, family income, professional category, work shift, professional experience, sleep duration, or any other variables assessed.

Finally, the correlation analysis (Table 4) indicated a significant negative association between absences due to musculoskeletal symptoms and the dimensions of vigor (r = −0.248; *p* < 0.05) and overall engagement score (r = −0.257; *p* < 0.05). Although most correlations did not reach significance, there was a consistent trend toward an inverse relationship between well-being indicators (dedication and absorption) and musculoskeletal symptoms, whereas burnout and occupational stress showed positive, though non-significant, associations. Although statistically significant, the correlations between vigor, overall engagement, and absenteeism were weak (r < 0.30) and should be interpreted with caution.

## 4. Discussion

The findings of this study reinforce the complexity of the physical and mental health repercussions of the COVID-19 pandemic on nursing professionals. Unlike other national and international investigations [17,28] that identified a high prevalence of compassion fatigue—including recent evidence demonstrating its persistence among frontline nurses during and after the pandemic, such as the findings of Pergol-Metko et al. [35], who observed significant levels of compassion fatigue, burnout, and the moderating effect of perceived social support among Polish hospital nurses—no cases of compassion fatigue were detected in our sample.

This result is consistent with those of a study conducted in a Brazilian university hospital during the pandemic, which likewise did not identify compassion fatigue profiles among frontline nurses despite substantial physical and emotional overload. In that investigation, high levels of dedication and vigor were associated with more favorable emotional well-being, though causality could not be established [6]. Similar patterns of preserved emotional resources, even under intense pandemic exposure, have also been described internationally, particularly among ICU nurses, for whom work engagement contributed to improved psychological adjustment [5].

These findings should be interpreted within the epidemiological context of the data collection period (February–April 2022), when the region was still experiencing high COVID-19 transmission and HU-FURG operated with expanded intermediate and intensive care capacity dedicated to pandemic response.

Although significant proportions of professionals exhibited high burnout and secondary traumatic stress, these dimensions did not occur simultaneously—a necessary condition for compassion fatigue. This suggests the presence of contextual factors mitigating emotional vulnerability, possibly related to professional maturity, length of experience, team cohesion, and organizational support [5,15,17].

These constructs, however, represent distinct dimensions: occupational stress measures perceived situational tension; burnout reflects chronic emotional exhaustion; secondary traumatic stress captures trauma-related symptoms resulting from repeated exposure to suffering; and compassion fatigue emerges only when burnout and secondary traumatic stress are simultaneously high.

The coexistence of a very low prevalence of occupational stress with notable levels of burnout and secondary traumatic stress warrants conceptual clarification. Occupational stress (WSS) evaluates perceived situational strain linked to daily work conditions [25], whereas burnout and secondary traumatic stress capture chronic and cumulative emotional responses to overload and exposure to suffering [26]. Thus, a professional may not perceive immediate or situational stress while still experiencing emotional exhaustion or trauma-related symptoms accumulated during prolonged exposure to critical events. In our sample, burnout and secondary traumatic stress did not overlap at high levels, explaining the absence of compassion fatigue despite signs of emotional vulnerability.

Although burnout and secondary traumatic stress share overlapping elements, such as emotional exhaustion and exposure to suffering, they remain distinct constructs in the ProQOL model [26]. Burnout reflects chronic exhaustion associated with workload and organizational demands, whereas secondary traumatic stress involves symptoms resembling post-traumatic responses triggered by repeated exposure to patient suffering [36,37]. Compassion fatigue arises only when both constructs reach high levels simultaneously, a pattern not observed in this sample. This conceptual distinction clarifies why compassion fatigue was absent despite the presence of emotional symptomatology [38].

Even without cases of compassion fatigue, the presence of high levels of burnout and secondary traumatic stress indicates emotional vulnerability among professionals, consistent with evidence of the cumulative impact of prolonged exposure to critical care situations during the pandemic [14,39].

The combination of physical and psychological demands, together with the risk of infection and the daily confrontation with suffering and death, can generate progressive exhaustion, even when mitigated by institutional or individual coping strategies. These elements help explain the coexistence, in this study, of signs of emotional exhaustion alongside very high levels of work engagement, especially dedication. This finding reinforces national evidence showing that even in the presence of anxiety and depression, nursing professionals may maintain dedication and a positive bond with their work, expressing resilience and motivation in the face of adversity [8,28,40]. Consistent findings have been reported in hospital settings in Saudi Arabia, where nurses demonstrated high levels of vigor and dedication influenced by professional experience and organizational factors [41].

The low prevalence of occupational stress may also be understood in light of the high engagement levels reported. Even in scenarios marked by long working hours, overload, and care pressure, strong involvement with work and awareness of its impact on patient care have been associated with lower perceived occupational stress [2,28]. Similar results have been found in health training programs, where higher levels of vigor and dedication were associated with greater satisfaction and lower turnover intention, suggesting that engagement functions as a psychosocial resource that supports job retention and quality of care [6].

These findings also align with evidence from Primary Health Care settings, where nurses tended to show strong career commitment, particularly through identification with their professional trajectory [42]. This suggests that positive ties to work and career, anchored in resilience and professional identity, may contribute to lower occupational stress and emotional exhaustion. Stability and opportunities for career development may reinforce not only job satisfaction and retention but also the preservation of health and well-being.

The very high levels of engagement observed in this study suggest greater dedication and vigor even under challenging conditions, indicating a potential association with better adaptation to work demands. Previous studies indicate that engagement can be strengthened by supportive organizational conditions and social support [43]. Nonetheless, a paradox must be considered: engagement, although beneficial, can lead to overcommitment when not accompanied by organizational support and adequate recovery mechanisms, potentially contributing to overload and exhaustion [39,44,45]. Recent analyses of mental health workers during the pandemic similarly showed that engagement may coexist with stress and overload in prolonged emergency contexts [46].

In the physical domain, the results revealed a high prevalence of musculoskeletal symptoms, especially in the lumbar region, consistent with evidence identifying nursing as one of the occupations most exposed to musculoskeletal disorders [7,12]. Recent studies further show that musculoskeletal pain contributes to psychological symptoms such as fatigue, anxiety, and depression, reinforcing its multidimensional impact [47].

Postural overload, repetitive movements, and continuous physical effort were intensified during the pandemic, increasing absenteeism and reducing quality of life. These conditions stem from the nature of nursing work—repetitive tasks, awkward postures, physical exertion, and long hours of standing—exacerbated during pandemic-related overload [10,12]. International evidence recognizes WMSDs as a leading cause of work disability, with implications for workforce health and the sustainability of health services [8,47]. Addressing these challenges requires ergonomic interventions, posture training, and workplace health promotion programs to improve working conditions and preserve the physical health of nursing professionals.

Beyond physical harm, musculoskeletal illness also influences emotional well-being, as professionals on leave frequently report feelings of guilt—a phenomenon documented in other health emergencies [48]. This overlap between physical and psychological suffering illustrates the multidimensional nature of overload experienced by nurses [47].

The results also reflect structural inequalities. Women reported more acute symptoms than men, consistent with evidence linking gender disparities to heavier workloads, lower recognition, and greater exposure to inappropriate postures [49,50]. These findings echo broader structural analyses showing that nursing—predominantly composed of women in Brazil—is a profession marked by social and economic vulnerabilities, exacerbated in crisis contexts [34].

Physical activity emerged as a protective factor against chronic symptoms, supporting evidence on the value of workplace health promotion [51,52]. This underscores the importance of encouraging physical activity adapted to the demands of nursing work.

Employment status was also associated with absences, with CLT employees reporting a higher mean number of absences than statutory workers. This may reflect differences in workload distribution, job demands, or organizational policies regarding authorized leave. Statutory workers may benefit from more stable trajectories, predictable schedules, or stronger institutional support, whereas CLT workers may face greater physical burden or less favorable working conditions during the pandemic [53,54].

Regarding psychological variables, a significant negative correlation was observed between vigor and overall engagement and the number of absences. Although statistically significant, these correlations were weak (r ≈ −0.25), indicating a small magnitude of association. This finding is consistent with studies showing that psychological well-being is associated with greater resilience to work demands and reduced perception of musculoskeletal pain [23,47]. Although burnout and occupational stress did not show significant associations with chronic symptoms, the results suggest that emotional exhaustion may influence the perception of physical symptoms, warranting longitudinal investigations [51,52].

In summary, this study advances existing evidence by integrating physical and psychological dimensions of occupational health within a single analytical model. Work engagement was associated with more favorable psychological indicators, although the study design does not allow conclusions about buffering effects. Unlike prior research examining these aspects separately, our results highlight engagement as a multidimensional construct associated with lower stress-related outcomes and fewer musculoskeletal symptoms, even in resource-constrained settings—but without implying a causal mitigating effect. This interpretation aligns with recent international studies demonstrating that engagement remains a relevant psychosocial resource for sustaining well-being and performance among nurses in diverse contexts during and after the pandemic [5,41,46].

These findings provide evidence to inform organizational and policy interventions that promote safe and supportive work environments, incorporating ergonomic measures, mental health programs, and engagement-based strategies to strengthen workforce resilience and retention in healthcare systems.

### 4.1. Limitations and Contributions

This study has several limitations, including its cross-sectional design, which does not allow for the establishment of causal relationships. Thus, all findings represent associations rather than causal effects. In addition, the results reflect the specific context of a single hospital in southern Brazil, which may limit their generalizability to other settings. The healthy worker effect may also have led to an underestimation of physical and emotional symptoms among professionals who remained active during the pandemic. The use of self-administered instruments increases the likelihood of social desirability bias, particularly in responses related to stress, burnout, and absenteeism. The limited sample size further reduced the statistical power to detect more subtle associations, and 20.8% of participants did not report their age, which may have restricted analyses involving age-related differences.

Despite these limitations, the study advances the field by adopting a multidimensional approach that integrates physical and psychological aspects into a comprehensive analysis of nursing professionals’ health. The identification of risk factors, such as female sex, and protective factors, such as physical activity and work engagement, contributes to the refinement of targeted intervention strategies. The results support the implementation of institutional policies and contribute to the international debate on working conditions in crisis contexts, reinforcing the need for structural measures that ensure health, dignity, and recognition for professionals who sustain health systems. Future longitudinal studies are needed to confirm the causal direction of the associations identified.

### 4.2. Reflections and Planning

The findings of this study underscore the need for interventions focused on the physical and emotional well-being of nursing professionals. To reduce occupational overload, it is essential to implement institutional policies that address ergonomic work organization, stress management, the promotion of regular physical activity, psychological support, and training in resilience and self-care, as well as measures that strengthen stability and professional recognition. Targeted actions for more vulnerable groups, such as women, are also strategic for mitigating musculoskeletal symptoms and promoting functional capacity and continuity of care [10,42,48,55].

These strategies should integrate physical and emotional dimensions, linking ergonomic and disease-prevention programs with psychological support and socio-emotional skills development. In addition, the creation of institutional policies aligned with recommendations from the World Health Organization and the International Labour Organization is crucial to protect workers during health crises. Investing in safe working conditions and preventive programs not only promotes the well-being of professionals but also ensures the quality of care delivered and the sustainability of health systems.

## 5. Conclusions

This study revealed the coexistence of musculoskeletal symptoms and emotional strain among frontline nursing professionals during the COVID-19 pandemic, without evidence of compassion fatigue. Although substantial proportions of professionals exhibited high levels of burnout and secondary traumatic stress, these conditions did not occur simultaneously. Instead, high engagement, particularly dedication and vigor, emerged as a possible protective factor against occupational stress.

Musculoskeletal symptoms were especially prevalent among women and among those not engaging in physical activity, reinforcing the importance of ergonomic strategies and health promotion initiatives. The originality of these findings lies in the paradoxical scenario of high engagement coexisting with emotional vulnerability, but without compassion fatigue, suggesting that resilience and professional identity may function as protective resources.

Maintaining the health of nursing professionals requires multidimensional strategies that integrate physical and psychological care, including ergonomic programs, emotional support, stress management, and the promotion of physical activity. Strengthening institutional policies that value and protect the workforce is essential to ensure safe, sustainable, and resilient health systems in crisis contexts.

## Figures and Tables

**Table 1:** Assessment of nursing professionals’ levels of engagement at work.

Dimension	Min	Max	Median	Mean ± SD	95% CI	Interpretation
Dedication	3.0	6.0	5.3	5.3 ± 0.7	5.1–5.4	Very High
Absorption	2.0	6.0	4.7	4.5 ± 0.9	4.3–4.8	High
Vigor	2.7	6.0	5.0	4.9 ± 0.8	4.7–5.1	Very High
Overall Score	3.0	6.0	5.0	4.9 ± 0.1	4.7–5.1	Very High

Min: minimum, Max: maximum, SD: standard deviation, 95% CI: 95% confidence interval.

**Table 2:** Frequency of musculoskeletal symptoms in nursing professionals, according to anatomical region.

Anatomical Region	Musculoskeletal Symptoms Past 7 Days		Musculoskeletal Symptoms Past 12 Months		Absences Due to Musculoskeletal Symptoms-Past 12 Months	
	n	%	n	%	n	%
Lower back	28	37.3	45	60	20	26.3
Neck	22	28.9	37	48.1	10	13.2
Shoulder	24	31.2	34	44.2	13	17.3
Back	18	24.0	29	39.2	9	12
Wrists/hands/fingers	9	12.0	18	24.0	10	13.3
Ankle/feet	7	9.3	14	18.7	8	10.7
Knees	5	6.7	13	17.3	7	9.3
Hips/thighs	4	5.3	11	14.7	6	8.0
Forearm	6	7.9	8	10.7	4	5.3
Elbows	2	2.7	7	9.2	2	2.7

**Table 3:** Association between sociodemographic/occupational variables and acute, chronic, and musculoskeletal symptom-related absences among nursing professionals.

Sociodemographic and Occupational Variables *	n	%	Acute Musculoskeletal Symptoms	Chronic Musculoskeletal Symptoms	Musculoskeletal Symptom-Related Absences
			Mean (SD) **	Mean (SD) **	Mean (SD) **
Sex					
Male	24	31.2	0.68 (1.13)	2.26 (2.34)	0.91 (0.87)
Female	53	68.8	1.94 (1.56)	2.96 (1.96)	1.10 (1.62)
*p*-value ***			<0.001	0.126	0.604
Age					
26–35 years	18	29.5	1.53 (1.28)	2.71 (1.96)	0.72 (1.02)
≥36 years	43	48.1	1.39 (1.64)	2.69 (2.22)	0.97 (1.50)
*p*-value ***			0.762	0.983	0.522
Family Income ****					
2–5 minimum wages	44	59.5	1.41 (1.63)	2.60 (2.19)	1.02 (1.47)
6–10 minimum wages	30	40.5	1.65 (1.39)	2.93 (1.98)	1.08 (1.38)
*p*-value ***			0.537	0.529	0.881
Professional Category					
Nurse	23	29.9	1.55 (1.54)	2.67 (1.91)	0.94 (1.17)
Nursing Assistant/Technician	54	70.1	1.54 (1.57)	2.76 (2.19)	1.07 (1.52)
*p*-value ***			0.981	0.866	0.740
Type of Employment					
Consolidation of Labor Laws (CLT)	12	15.8	2.00 (2.11)	3.10 (2.77)	1.90 (1.73)
Statutory	64	84.2	1.49 (1.44)	2.72 (1.98)	0.92 (1.34)
*p*-value ***			0.340	0.594	0.043
Work Shift					
Full-time Day	35	45.4	2.43 (1.27)	2.71 (1.69)	1.43 (1.40)
Night	41	53.2	1.47 (1.55)	2.78 (2.14)	1.02 (1.44)
*p*-value ***			0.120	0.939	0.473
Professional Experience					
<10 years	28	37.3	0 (0.0)	1.50 (2.38)	0.25 (0.50)
≥10 years	47	62.7	1.66 (1.54)	2.83 (2.08)	1.04 (1.43)
*p*-value ***			0.036	0.220	0.274
Daily Sleep Duration					
<6 h	24	31.2	1.00 (0.82)	2.52 (2.04)	0.81 (1.08)
6–8 h	53	68.8	1.75 (1.71)	2.82 (2.13)	1.14 (1.55)
*p*-value ***			0.073	0.590	0.380
Engages in Physical Activity					
Yes	32	41.6	1.20 (1.47)	2.19 (2.04)	0.84 (1.12)
No	45	58.4	1.80 (1.57)	3.15 (2.07)	1.20 (1.62)
*p*-value ***			0.109	0.028	0.298

* Percentages refer to valid responses for each variable. ** SD = Standard Deviation. *** *p*-values obtained using the Mann–Whitney U test (two groups) or Kruskal–Wallis test (more than two groups). **** Minimum wage: R$ 1212.00/USD 234.86 (1 USD = R$ 5.1604).

**Table 4:** Correlation between psychological variables and musculoskeletal symptoms in nursing professionals.

Psychological Variables	Acute Musculoskeletal Symptoms	Chronic Musculoskeletal Symptoms	Musculoskeletal Symptom-Related Absences
Compassion Satisfaction	0.0562	−0.1211	−0.1449
Burnout	0.0713	−0.008	0.1627
Secondary Traumatic Stress	0.0812	0.1388	0.1164
Dedication	−0.1428	−0.0545	−0.1864
Absorption	−0.0209	−0.002	−0.1915
Vigor	−0.1645	−0.0868	−0.2482 *
Overall Score	−0.131	−0.0606	−0.2571 *
Occupational Stress	0.0248	0.1279	−0.1004

* Significant correlations (*p* < 0.05).

## Data Availability

The data presented in this study are available on request from the corresponding author due to ethical approval requirements.

## Abbreviations

The following abbreviations are used in this manuscript:

95% CI	95% Confidence Interval
CAAE	Certificate of Presentation and Ethical Appreciation
HU/FURG	Dr. Miguel Riet Correa Júnior University Hospital of the Federal University of Rio Grande
Max	Maximum
Md	Median
Min	Minimum
NMSQ	Nordic Musculoskeletal Symptom Questionnaire
ProQOL-BR	Professional Quality of Life Scale
sd	Standard Deviation
SPSS	Statistical Package for Social Sciences^®^
UWES-9	Utrecht Work Engagement Scale
WMSDs	Work-Related Musculoskeletal Disorders
WSS	Work Stress Scale
